# Adoption of artificial intelligence in healthcare: survey of health system priorities, successes, and challenges

**DOI:** 10.1093/jamia/ocaf065

**Published:** 2025-05-05

**Authors:** Eric G Poon, Christy Harris Lemak, Juan C Rojas, Janet Guptill, David Classen

**Affiliations:** Duke University Health System, Durham, NC, United States; Department of Medicine, Duke University School of Medicine, Durham, NC, United States; Department of Biostatistics and Bioinformatics, Duke University School of Medicine, Durham, NC, United States; Department of Health Services Administration, University of Alabama at Birmingham, Birmingham, AL, United States; Scottsdale Institute, Scottsdale, AZ, United States; Rush University System for Health, Chicago, IL, United States; Scottsdale Institute, Scottsdale, AZ, United States; Division of Epidemiology, University of Utah School of Medicine, Salt Lake City, UT, United States

**Keywords:** artificial intelligence, generative AI, responsible AI, technology adoption, AI safety

## Abstract

**Importance:**

The US healthcare system faces significant challenges, including clinician burnout, operational inefficiencies, and concerns about patient safety. Artificial intelligence (AI), particularly generative AI, has the potential to address these challenges, but its adoption, effectiveness, and barriers to implementation are not well understood.

**Objective:**

To evaluate the current state of AI adoption in US healthcare systems, assess successes and barriers to implementation during the early generative AI era.

**Design, setting, and participants:**

This cross-sectional survey was conducted in Fall 2024, and included 67 health systems members of the Scottsdale Institute, a collaborative of US non-profit healthcare organizations. Forty-three health systems completed the survey (64% response rate). Respondents provided data on the deployment status and perceived success of 37 AI use cases across 10 categories.

**Main outcomes and measures:**

The primary outcomes were the extent of AI use case development, piloting, or deployment, the degree of reported success for AI use cases, and the most significant barriers to adoption.

**Results:**

Across the 43 responding health systems, AI adoption and perceptions of success varied significantly. Ambient Notes, a generative AI tool for clinical documentation, was the only use case with 100% of respondents reporting adoption activities, and 53% reported a high degree of success with using AI for Clinical Documentation. Imaging and radiology emerged as the most widely deployed clinical AI use case, with 90% of organizations reporting at least partial deployment, although successes with diagnostic use cases were limited. Similarly, many organizations have deployed AI for clinical risk stratification such as early sepsis detection, but only 38% report high success in this area. Immature AI tools were identified a significant barrier to adoption, cited by 77% of respondents, followed by financial concerns (47%) and regulatory uncertainty (40%).

**Conclusions and relevance:**

Ambient Notes is rapidly advancing in US healthcare systems and demonstrating early success. Other AI use cases show varying degrees of adoption and success, constrained by barriers such as immature AI tools, financial concerns, and regulatory uncertainty. Addressing these challenges through robust evaluations, shared strategies, and governance models will be essential to ensure effective integration and adoption of AI into healthcare practice.

## Introduction

As the US healthcare system grapples with significant challenges in quality, affordability, and labor shortages, Artificial Intelligence (AI) holds promise for transforming the delivery system to become safer, more effective, less wasteful, and more patient-centered.^1^^,^^2^ AI has already demonstrated success in preventing sepsis,^3^ improving diagnostic accuracy in radiology and pathology,^4–6^ and reducing clinicians’ documentation burden.^7–12^ Driven by recent advances in generative AI, investment in health AI continues to grow, with over $30B invested into the healthcare AI companies in the past 3 years.^13^ This has resulted in a proliferation of solutions that could provide relief to the strained healthcare delivery system.

While AI has been integrated into many aspects of modern life, its extent of use in healthcare delivery remains unclear. The medical literature showcases many examples, but the vast majority have only been demonstrated on select organizations and/or patient populations. As with all forms of innovation, the path to widespread adoption and societal impact is long and by no means certain.^14^^,^^15^ Adoption of AI in healthcare likely faces unique sociological and technical challenges.^16^ As such, an assessment of the current AI use and impact in healthcare may inform innovators, technology vendors, policy makers, and organizational decision makers.

Factors that influence technology adoption are dynamic. Previous studies that have evaluated AI deployment in US healthcare organizations^17–20^ are limited in scope and took place before current generative AI has significantly impacted the healthcare industry. It remains unclear how traditional AI, which focuses on predicting clinical or operational outcomes, is being deployed alongside more advanced AI tools that interpret and generate human language and images. In addition, while previous studies have identified various barriers to utilizing AI in healthcare, their relative importance remains unclear.^17^^,^^18^ In light of the new-found promise and enthusiasm surrounding generative AI, our team aimed to assess AI adoption early in the generative AI era, identify the most significant barriers faced by healthcare organizations, and highlight initial examples of successes.

## Methods

### Instrument development

Our team of healthcare, medical informatics, and organizational management experts developed a survey instrument to (1) assess the state of and drivers behind AI adoption in major US health systems, (2) evaluate successes and barriers for AI, and (3) identify candidates for (future) in-depth case studies.

To develop the survey instrument, we reviewed the literature and analyzed transcripts from various industry meetings, including prior Scottsdale Institute (SI)'s AI surveys^17^^,^^18^ and SI membership AI conferences between Spring 2023 and Spring 2024. First, we identified six key themes related to why health systems may be developing and deploying AI tools: Patient Safety/Quality, Caregiver Burden/Satisfaction, Margin Improvement/Financial, Workflow Efficiency/Productivity, Patient/Consumer Experience, and Market Share/Competitiveness. We asked respondents to rank these 6 organizational goals. We also compiled AI use cases from literature, conference transcripts, and past research,^18^ identifying 37 use cases in 10 categories related to clinical care, healthcare operations, analytics and research (Appendix S1).

Respondents reported the status of AI adoption in their organizations–developing, piloting, partially deploying, or fully deployed—for each use case. Respondents could also add unlisted use cases via free text. We asked respondents about their perceived degree of success of AI use cases in each of the 10 categories. Based on results of our literature search and prior work, we identified 9 common metrics used to assess the performance of AI tools, and we asked respondents to report how frequently they used each of them. Six key barriers to adopting AI were also identified: financial concerns, regulatory uncertainty, lack of leadership support, low clinician adoption, insufficient expertise or technology, and immature AI tools. Respondents were asked to prioritize these barriers.

To further refine the instrument, we conducted iterative reviews and pilot testing with a small group of health system leaders. This process allowed us to assess the clarity, relevance, and comprehensiveness of the questions, ensuring that they effectively captured meaningful insights. The finalized survey instrument appears in Appendix S2. We used the American Association for Public Opinion Research (AAPOR) Best Practices for Survey Research^21^ to ensure the accuracy and reliability of our findings.

### Participant recruitment

The organizations included in the invitation were members of SI, a not-for-profit organization that assists its 67 US non-profit health systems in accomplishing quality improvement, organizational efficiency, patient safety and transformation through information technology. Invitations were sent to all 67 SI members’ Chief Medical Information Officers, Chief Information/Digital Officers, and/or Chief AI Officers via email from September 27 to October 20, 2024. Each health system was asked to provide one single response via the survey software SurveyMonkey. The Institution Review Board (IRB) at the University of Alabama at Birmingham approved the study (IRB-300009793).

### Data analysis

The research team aggregated survey data using Microsoft Excel. Characteristics of health systems represented by survey respondents and non-survey respondents were compared with those of US health systems using online statistical calculators for Chi-Squared test and Fischer’s Exact test.

## Results

Of 67 total health systems surveyed, 46 answered at least one question and 43 completed the entire survey (64% survey completion rate). The 3 health systems that attempted but did not complete the survey only responded to the opening question and were discarded from further analysis. Characteristics of (completed) survey respondents and non-respondents in terms of size by net patient revenue (NPR), size by number of hospitals, teaching status, and geography (as sourced from Definitive Healthcare^22^) are summarized in Table 1. There was no statistically significant difference between survey respondents versus non-respondents, except a higher proportion of our respondents had an NPR between $5B and $9.9B compared to non-respondents. Compared to all US healthcare systems, survey respondents were more heavily represented in the 2 middle categories for NPR ($1-$4.9 billion and $5-9.9 billion). The most common title of the respondents were Chief [Medical | Health | Clinical] Information Officers (50%, *n* = 23), while 13% (*n* = 6) held the Chief [Information | Digital] Officer title, and another 13% (*n* = 6) held the Chief [Analytics | AI] Officer or Chief Data Scientist title.

**Table 1.** Characteristics of US hospitals, organizations invited to participate in the survey, and responding organizations.

	US health systems *n*(%)	SI members				US health systems vs survey respondents (*P* value)
		Overall *n*(%)	Survey respondents *n*(%)	Survey non-respondents *n*(%)	Respondents vs non-respondents (*P* value)	
Size—NPR					.04^a^	<.01^b^
Under $1 billion	53 (26%)	1 (1%)	1 (2%)	0 (0%)		
$1 billion-$4.9 billion	97 (48%)	43 (64%)	25 (58%)	15 (71%)		
$5 billion-$9.9 billion	36 (18%)	17 (25%)	15 (35%)	2 (10%)		
$10 billion and above	17 (8%)	6 (9%)	2 (5%)	4 (19%)		
Size—number of hospitals					.79^b^	.93^b^
4 or fewer hospitals	62 (31%)	16 (24%)	12 (28%)	4 (19%)		
5-10 hospitals	57 (28%)	23 (34%)	14 (33%)	6 (29%)		
11-19 hospitals	41 (20%)	15 (22%)	9 (21%)	6 (29%)		
20+ hospitals	43 (21%)	13 (19%)	8 (19%)	5 (24%)		
Teaching status					.07^a^	.37^b^
Major teaching	122 (60%)	51 (76%)	29 (67%)	18 (90%)		
Non-major teaching	81 (40%)	16 (24%)	14 (33%)	2 (10%)		
Geographic region					.16^a^	.15^b^
Northeast	37 (18%)	6 (9%)	3 (7%)	2 (10%)		
South	72 (35%)	21 (31%)	13 (30%)	8 (38%)		
Midwest	55 (27%)	27 (40%)	15 (35%)	10 (48%)		
West	38 (19%)	13 (19%)	12 (28%)	1 (5%)		
US Territory	1 (0%)	0 (0%)	0 (0%)	0 (0%)		

a Fisher’s exact test (http://vassarstats.net/fisher2x4.html).

b Chi-squared test (https://www.socscistatistics.com/tests/).

### Survey results

**Figure 1.**
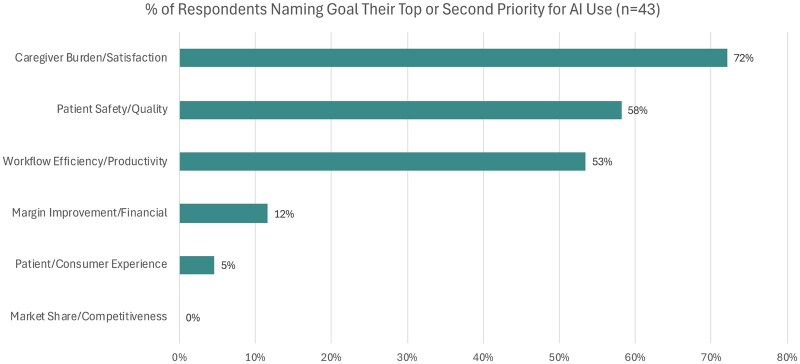
Key goals for deploying AI. Proportion of respondents citing each goal as the highest or second-highest priority.

Among the key organizational goals for deploying AI, the most cited priority was reducing caregiver burden and improving satisfaction, with 72% (*n* = 31) of organizations ranking it as one of their top two goals. Both patient safety/quality and workflow efficiency/productivity followed closely, with 56% (*n* = 25) and 53% (*n* = 23) of organizations identifying them as their top two priorities respectively. Margin improvement/financial, patient/consumer experience, and market share/competitiveness were infrequently cited as the top 2 priorities (Figure 1). The priorities for respondents with NPR greater than $5B (*n* = 17) were statistically similar to those from health systems with NPR less than $5B (*n* = 26).

**Figure 2.**
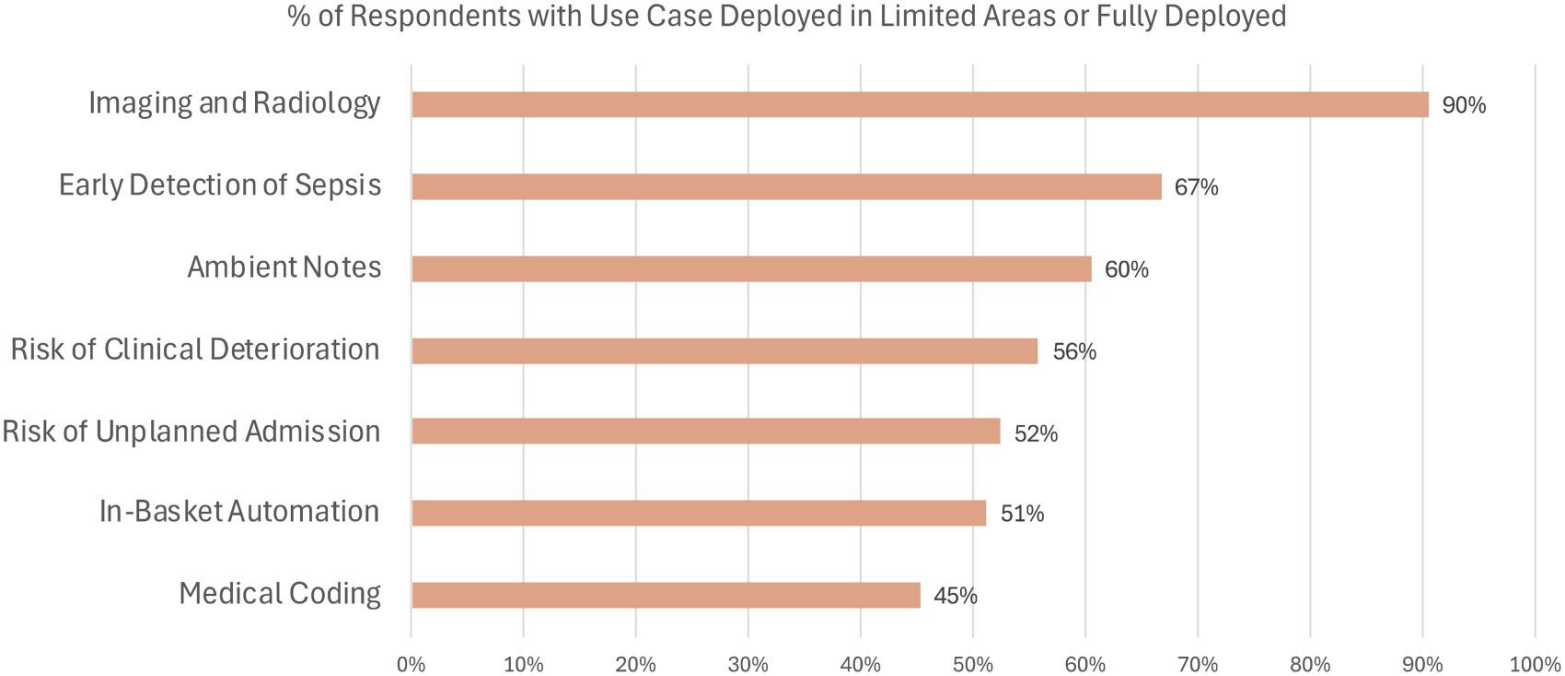
Most commonly deployed AI use cases. Proportion of respondents who have deployed each use case in at least limited areas.

Health systems demonstrated significant variation in the extent of AI use case development and deployment, as illustrated in Table 2. Among the most commonly deployed use cases, 90% (*n* = 38) of respondents reported deploying AI for Imaging and Radiology in at least limited areas. This was followed by early detection of sepsis (67%, *n* = 27), ambient notes (60%, *n* = 26), risk of clinical deterioration (56%, *n* = 24), predicting the risk unplanned readmission (52%, *n* = 22), and in-basket automation (51%, *n* = 22) (Figure 2). Among the 38 organizations that have deployed the Imaging and Radiology use case in at least limited areas, 17 reported full deployment. Notably, while only 14% (*n* = 6) of organizations have fully deployed Ambient Notes and another 47% (*n* = 20) have implemented it in limited areas, 100% of respondents have at least begun developing or piloting this use case, which transforms conversations between the clinician and the patient into draft clinical documentation—this degree of uniform early adoption was not seen with any of the other 36 use cases.

**Table 2.** AI uses cases and their implementation stage.

AI use case category	AI use case	No activity *n* (%)	Developing or piloting *n* (%)	Deploying in limited areas *n* (%)	Deployed fully *n* (%)
Clinical Documentation	Ambient Notes	0 (0%)	17 (40%)	**20 (47%)**	6 (14%)
	Ambient Nursing	13 (31%)	**28 (67%)**	1 (2%)	0 (0%)
	Drafting Care Plan Notes	12 (29%)	**28 (68%)**	1 (2%)	0 (0%)
	Abstracting Data for Clinical Registries	13 (31%)	**24 (57%)**	4 (10%)	1 (2%)
Clinical Chart Review	Inpatient Chart Summarization	9 (21%)	**30 (71%)**	3 (7%)	0 (0%)
	Ambulatory/Clinic Chart Summarization	8 (19%)	**31 (74%)**	3 (7%)	0 (0%)
Clinical Risk Stratification	Early Detection of Sepsis	5 (12%)	9 (21%)	8 (19%)	**20 (48%)**
	Risk of Unplanned Admission	8 (19%)	**12 (29%)**	10 (24%)	**12 (29%)**
	Risk of Clinical Deterioration	4 (9%)	15 (35%)	8 (19%)	**16 (37%)**
	Risk of Patient Falls	9 (21%)	**18 (43%)**	7 (17%)	8 (19%)
Diagnosis	Imaging and Radiology	2 (5%)	2 (5%)	**21 (50%)**	17 (40%)
	Digital Pathology	**16 (38%)**	**16 (38%)**	8 (19%)	2 (5%)
	Other Diagnostic Tools	11 (28%)	**14 (35%)**	12 (30%)	3 (8%)
Engaging Patients	In-Basket Automation	3 (7%)	**18 (42%)**	16 (37%)	6 (14%)
	Adjust Reading Levels	**23 (55%)**	18 (43%)	1 (2%)	0 (0%)
	Language Translation	**20 (48%)**	18 (43%)	4 (10%)	0 (0%)
	Remote Patient Monitoring	9 (21%)	**20 (47%)**	9 (21%)	5 (12%)
	Companion AI/Conversational Agents	10 (24%)	**23 (55%)**	7 (17%)	2 (5%)
	Care Navigation	15 (37%)	**25 (61%)**	1 (2%)	0 (0%)
Patient Access and Marketing	Predict Risk of Patient No Shows	10 (24%)	**19 (45%)**	7 (17%)	6 (14%)
	Automate Patient Visit Scheduling	16 (38%)	**22 (52%)**	3 (7%)	1 (2%)
	AI-Enabled Triage	20 (48%)	**21 (50%)**	1 (2%)	0 (0%)
Revenue Cycle	Medical Coding	7 (17%)	**16 (38%)**	9 (21%)	10 (24%)
	Automate Utilization Review	14 (34%)	**18 (44%)**	5 (12%)	4 (10%)
	Streamline Prior Authorization	9 (22%)	**24 (59%)**	2 (5%)	6 (15%)
Business Functions (not Rev Cycle)	Forecast Census and Staffing	8 (19%)	**20 (48%)**	5 (12%)	9 (21%)
	Optimize Patient Room Utilization	15 (36%)	**19 (45%)**	5 (12%)	3 (7%)
	Optimize Operating Room Utilization	11 (26%)	**17 (40%)**	8 (19%)	6 (14%)
	Supply Chain Tools	**18 (45%)**	16 (40%)	4 (10%)	2 (5%)
	Recruiting and Human Resource Tools	**16 (40%)**	15 (38%)	6 (15%)	3 (8%)
	Cash Forecasting (Treasury)	**25 (63%)**	12 (30%)	2 (5%)	1 (3%)
Automating Analytics	Data Analysis	3 (8%)	**23 (58%)**	12 (30%)	2 (5%)
	AI-enabled Computer Coding	7 (17%)	**23 (55%)**	9 (21%)	3 (7%)
Supporting Research	Clinical Trials Automation	**21 (50%)**	**21 (50%)**	0 (0%)	0 (0%)
	Drug Discovery	**25 (61%)**	16 (39%)	0 (0%)	0 (0%)
	Genetics, Genomics	19 (46%)	**22 (54%)**	0 (0%)	0 (0%)

Implementation stage with relative majority of responses **bolded** and highlighted in blue.

**Figure 3.**
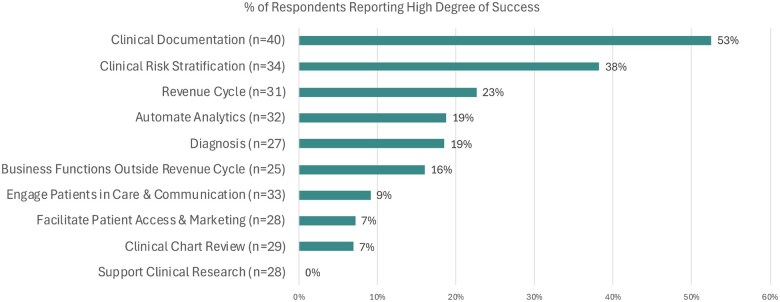
Successes reported among AI use case categories. Proportion of organizations reporting a high degree of success within each AI use case category, among those that have started developing, piloting, or deploying it.

Among organizations that have started developing, piloting and deploying the 10 use cases categories, 53% of respondents (*n* = 21) reported a “high degree of success” with use of AI in Clinical Documentation, while 38% (*n* = 13) reported that for Clinical Risk Stratification use cases, and only 23% (*n* = 7) reported that for Revenue Cycle use cases. Only 19% (*n* = 5) of respondents who have started developing, piloting or deploying AI use cases for Clinical Diagnosis reported a high degree of success (Figure 3). Regarding measures of success for AI tools, Use (Uptake) of the AI tool was always measured by 74% (*n* = 31) of the respondents, while Health Equity/Disparities was always measured only by 17% (*n* = 7). Of note, Health Equity/Disparities was rarely measured and not measured by 10% (*n* = 4) and 20% (*n* = 8) of the respondents, respectively (Appendix S3).

**Figure 4.**
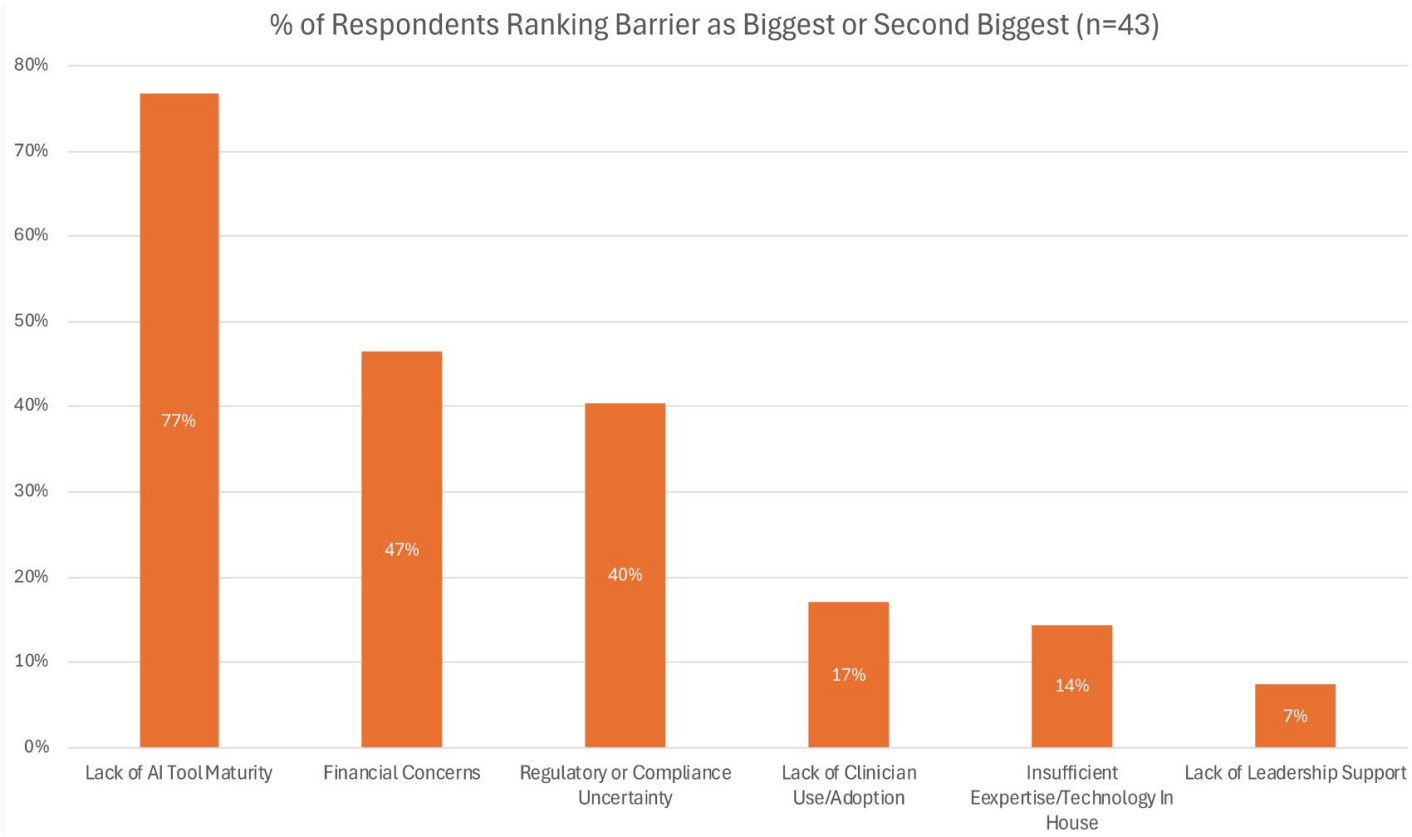
Key barriers to developing or deploying AI. Proportion of survey respondents (*n* = 43) who rated each barrier as the highest or second-biggest.

Among the 43 respondents, 77% (*n* = 33) identified lack of AI tool maturity as the biggest or second-biggest barrier to AI development or deployment. This was followed by financial concerns, reported by 47% (*n* = 20), and regulatory or compliance uncertainty, cited by 40% (*n* = 17). In contrast, lack of clinician use or adoption (17%, *n* = 7), insufficient in-house expertise or technology (14%, *n* = 6), and lack of leadership support (7%, *n* = 3) were infrequently reported as one of the top 2 barriers (Figure 4). The top barriers were statistically similar between respondents with NPR greater than $5B vs those with NPR less than $5B.

## Discussion

Our 2024 survey assessed the state of AI adoption in leading US health systems less than 2 years following the release of ChatGPT.^23^ All health system respondents reported adoption activities in Ambient Notes, with over half of them reporting a high degree of success in use of AI for Clinical Documentation, virtually all of which uses generative AI. The remainder of the AI use cases assessed showed variable degrees of adoption, with clinical risk stratification models showing moderate adoption and only 38% of respondents reporting a high degree of success. Similarly, while most organizations have deployed AI in imaging and radiology, only 19% of them report a high degree of success in this area.

Although Ambient Notes solutions were in development by vendors before ChatGPT, recent advances in Generative AI have accelerated their market readiness (Petro J, Chief Technology Officer, Nuance Communications. Personal Communication). In comparison to other use cases that have been in development for much longer, such as imaging and radiology, Ambient Notes using generative AI appears to have swiftly crossed the “chasm” that often hinders new technology products from progressing beyond the early adopters (typically technology enthusiasts and visionaries) to being embraced by the pragmatic early majority.^15^ This apparent rate of rapid adoption may in part be influenced by concerns about clinician burnout following the COVID-19 pandemic,^24^ as most respondents identified relieving caregiver burnout/improving satisfaction and improving workflow efficiency/productivity as primary reasons for adopting AI. This rate of adoption compares favorably to that of CTs and MRIs in the 1970s and 1980s, respectively,^25^ technologies that are now integral to the practice of medicine. However, the swift adoption of Ambient Notes raises concerns about potential unintended consequences, such as technology affordability, workforce readiness, trainee usage, and patient perception, which remain unresolved. In addition, given the evolving landscape of generative AI, future research is necessary to evaluate its impact on organizational and clinical outcomes, including clinician productivity, clinician retention, and patient satisfaction.

Adoption of AI outside of ambient notes is still uneven and incomplete. While several risk stratification AI tools were reported as deployed by about half of our respondents, only approximately one-third of those developing or deploying those use cases cited a high degree of success. Although some of these risk stratification models have been available at relatively modest costs to health systems for more than 5 years, their accuracy, at least for sepsis prediction, has been called into recent question by several recent studies.^26^ This aligns with the observation made by our respondents that AI tool immaturity is a leading barrier to AI adoption. Busy clinicians who may be too distracted or alert-fatigued to act on these risk predictions could also explain the modest success reported by institutions deploying them. Similarly, only 19% of institutions reported a high degree of success for AI used in clinical diagnosis, despite predictions dating back to 2016 of AI replacing radiologists^27^^,^^28^ and a steady stream of AI-based imaging solutions undergoing FDA approval.^29^ These data indicate the need for developers of imaging and radiology AI to further collaborate with imaging-based clinicians to improve their workflow and decision-making quality.^30^^,^^31^

Given the current immaturity of AI tools and the need to balance speed with safety, it is clear that the heightened expectations associated with AI^32^ will need to be managed through robust evaluations to ensure AI deployed in healthcare is safe, effective and equitable.^33^ This is particularly relevant given mounting cost pressures and uncertainties on health systems,^34^ which might further constrain resources necessary to deploy, test, and monitor AI and in turn jeopardize patient safety and the long-term value of AI. Fit-for-purpose and practical evaluations are necessary before an AI tool is deployed, during its implementation, and in an ongoing way to ensure that their benefits continue to justify ongoing costs and risks. In addition, local lessons learned throughout the life-cycle of AI deployment will be highly valuable given the varying adoption pace, implementation expertise, and sociotechnical factors across organizations.^35^ Traditional mechanisms for sharing these evaluations results and lessons learned, including academic journals and conferences may not meet the rapidly evolving needs of the health AI industry, and are subject to biases that tend to favor dissemination of positive findings. Proposals from Coalition for Health AI, VALID AI, Health AI Partnership and others^36^ are advocating for networks to distribute the evaluation work and disseminate the findings. While this healthcare provider-based approach will bring value, additional measures are needed. First, EHR and AI vendors should strive towards common deployment platforms to enable cost-effective AI tool integration at relevant workflow and decision-making points. This approach has proven effective with “traditional” tools such as medication decision support and standardized medical terminology. Without such an approach, individual clinicians might face multiple user-interfaces, adding cognitive load and potentially diminishing each other’s effectiveness. Second, local configuration decisions, usage policies, implementation strategies, evaluation tools, and user-support resources should be widely shared, perhaps utilizing Patient Safety Organizations to address confidentiality concerns. Such resources would accelerate the maturation speed for healthcare organizations and vendors. Third, professional and membership organizations should encourage members to publish results of programmatic outcome evaluations and case studies so that lessons learned could be applied at scale.

Many use cases assessed in this survey, particularly those in the fields of administration, patient engagement, marketing, and clinical research, are still in the early stages of adoption among our study population. With ongoing cost pressures facing health systems and continued investment in AI, widespread success may become achievable. However, the challenges faced by more mature clinical AI use cases will likely apply here as well. Therefore, it is crucial for national bodies focusing on responsible use of health AI to share their expertise with developers and implementers of these early use cases, many of whom may only be beginning to engage in the responsible AI conversation. Similarly, measures around equity and bias are not used by health systems consistently to monitor the performance of AI tools, in spite of strong recommendations by many health AI consortia to focus on algorithmic bias.^33^^,^^37^^,^^38^ This suggests the need for the dissemination of practical approaches that would allow local AI oversight bodies to monitor the differential performance and impact of AI tools across vulnerable populations.

Our study has several limitations. Our study presents only a snapshot of the state of AI adoption across 43 large health systems as of the Fall of 2024. Most SI members represent health systems with NPR of at least $1B and may have access to more resources than the average US health system. In addition, since SI members are actively engaged in quality improvement and learning, their level of AI adoption may be more advanced compared to their average peer. Nonetheless, their activities could indicate what other health systems might focus on in the near future, and challenges faced by our respondents may also affect others unless addressed at the national level. The preponderance of clinicians among our survey respondents could have led us to underestimate the adoption of AI in the business function and revenue cycle domains, and our findings may not generalize immediately to all healthcare organizations in the United States. However, the diverse geographical locations and types of organizations represented by our survey respondents enhance the relevance and applicability of our conclusions. Overall, our study underscores that AI tools, except for Ambient Notes, are deployed inconsistently and with varying degrees of success. As such, efforts to share evaluation results and lessons learned will contribute towards a common good across multiple stakeholders.

## Supplementary Material

ocaf065_Supplementary_Data

## Data Availability

Data available upon request.

## Findings

In this cross-sectional survey of 43 US health systems, all respondents reported activity related to Ambient Notes, a generative AI tool for clinical documentation. Other select AI use cases such as imaging/radiology and clinical risk stratification are adopted by many, though successes are modest. Immature AI tools and financial concerns were the most frequently cited barriers.

## Meaning

Generative AI tools like Ambient Notes show significant promise and rapid adoption is occurring. However, other AI tools continue to be adopted unevenly. Challenges such as AI tool immaturity and financial constraints must be overcome to ensure broad adoption and impact.
