# Supplementary material for: Adoption of artificial intelligence in healthcare: survey of health system priorities, successes, and challenges
Source: J Am Med Inform Assoc. 2025 May 5;32(7):1093–100. doi: 10.1093/jamia/ocaf065 (PMC12202002; doi:10.1093/jamia/ocaf065)

Appendix 1 – AI Use Cases Assessed In Survey. 37 AI use cases grouped under 10 categories

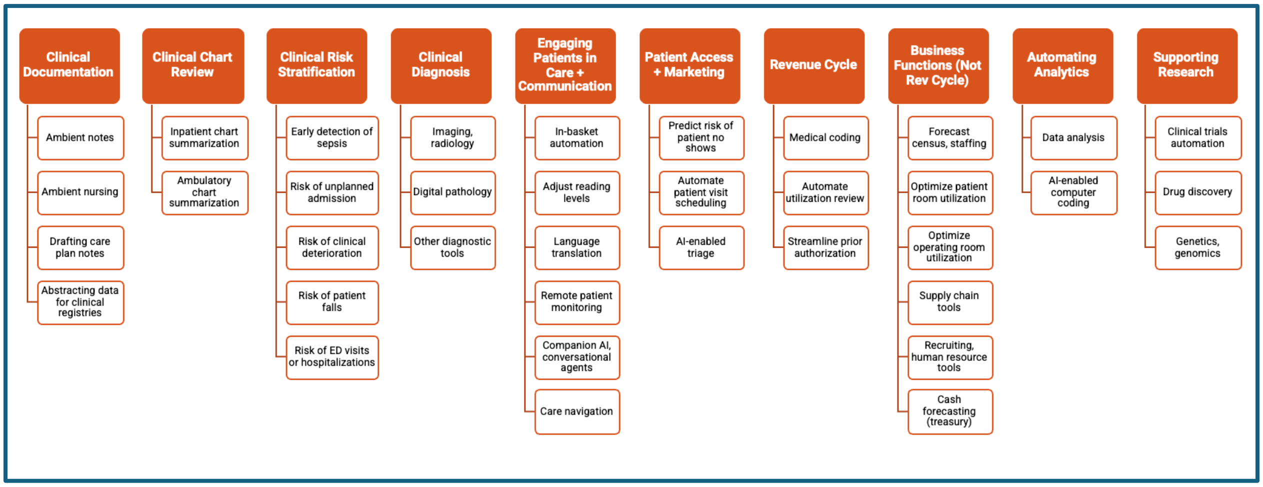

Appendix 2 – Survey Instrument

SCOTTSDALE INSTITUTE
COLLABORATION · EDUCATION · NETWORKING

AI Tools in Use in Scottsdale Institute Systems - Fall 2024

1. Thank You for Participating!

The Scottsdale Institute is surveying our members about how they are developing, piloting and deploying AI tools. We look forward to sharing the results with you.

Your survey responses will be aggregated in all analyses and reporting and your identity and your organization's identity will not be revealed beyond a list of those organizations participating in this survey.

We ask that each SI member system complete one survey. Please coordinate with your team and complete one survey. Thank you for your participation and valuable insights.

If you have used the hard copy survey to prepare, the survey should take about 10 minutes to complete.

2. Overall Use of AI Tools

3. Is your health system currently **developing, piloting, or using Artificial Intelligence (AI) tools**?

By AI tools, we mean any type of AI tool (e.g., predictive analytics, large language models, computer-assisted diagnosis, computer vision and the like).

- ◯ Not at this time.
- ◯ Yes, one or more AI tools is in development, pilot testing or deployment.

3. Main Goals for Using AI Tools

4. What are your health system's **main goals** for using AI tools?

Please slide each possible goal to rank them, with most important goals at the top.

≣	Patient Safety/Quality	☐ N/A
≣	Caregiver Burden/Satisfaction	☐ N/A
≣	Margin Improvement/Financial	☐ N/A
≣	Workflow Efficiency/Productivity	☐ N/A
≣	Patient/Consumer Experience	☐ N/A
≣	Market Share/Competitiveness	☐ N/A

4. AI Use Cases (Developing, Piloting or Deploying)

The next questions ask about AI tools in various categories (Clinical Documentation, Chart Review, Risk Stratification, Diagnoses, Engage Patients, Facilitate Access, Revenue Cycle, Other Business Functions, Automate Analyses, Support Clinical Research).

5. For each of the following AI tools related to **Clinical Documentation**, please indicate the extent to which they are being developed, piloted or deployed in your health system.

	Not at All	Developing (including research)	Piloting	Deploying in Limited Areas	Deployed Fully
Ambient Notes	☐	☐	☐	☐	☐
Ambient Nursing	☐	☐	☐	☐	☐
Drafting Care Plan Notes	☐	☐	☐	☐	☐
Abstracting Data for Clinical Registries	☐	☐	☐	☐	☐

Other (please specify)

6. For each of the following AI tools related to **Clinical Chart Review,** please indicate the extent to which they are being developed, piloted or deployed in your health system.

	Not at All	Developing (including research)	Piloting	Deploying in Limited Areas	Deployed Fully
Inpatient Chart Summarization	☐	☐	☐	☐	☐
Ambulatory/Clinic Chart Summarization	☐	☐	☐	☐	☐

List Other AI Tools Related to Clinical Documentation and indicate Developing, Piloting, or Deploying:

7. For each of the following AI tools related to **Clinical Risk Stratification**, please indicate the extent to which they are being developed, piloted or deployed in your health system.

	Not at All	Developing (including research)	Piloting	Deploying in Limited Areas	Deployed Fully
Early Detection of Sepsis	☐	☐	☐	☐	☐
Risk of Unplanned Admission	☐	☐	☐	☐	☐
Risk of Clinical Deteroriation	☐	☐	☐	☐	☐
Risk of Patient Falls	☐	☐	☐	☐	☐
Risk of ED Visits or Hospitalizations	☐	☐	☐	☐	☐

List Other AI Tools Related to Clinical Risk Stratification and indicate Developing, Piloting, or Deploying:

8. For each of the following AI tools related to **Clinical Diagnosis,** please indicate the extent to which they are being developed, piloted or deployed in your health system.

	Not at All	Developing (including research)	Piloting	Deploying in Limited Areas	Deployed Fully
Imaging and Radiology Tools	☐	☐	☐	☐	☐
Digital Pathology	☐	☐	☐	☐	☐
Other Diagnostic AI Tools	☐	☐	☐	☐	☐

List Other AI Tools Related to Clinical Diagnosis and indicate Developing, Piloting, or Deploying:

9. For each of the following AI tools related to **Engaging Patients in Care or Communication**, please indicate the extent to which they are being developed, piloted or deployed in your health system.

	Not at All	Developing (including research)	Piloting	Deploying in Limited Area(s)	Deployed Fully
In-Basket Automation	☐	☐	☐	☐	☐
Adjust Reading Levels	☐	☐	☐	☐	☐
Language Translation	☐	☐	☐	☐	☐
Remote Patient Monitoring	☐	☐	☐	☐	☐
Companion AI/Conversational Agents	☐	☐	☐	☐	☐
Care Navigation	☐	☐	☐	☐	☐

List Other AI Tools Related to Engaging Patients and indicate Developing, Piloting, or Deploying:

10. For each of the following AI tools related to **Patient Access and Marketing**, please indicate the extent to which they are being developed, piloted or deployed in your health system.

	Not at All	Developing (including research)	Piloting	Deploying in Limited Area(s)	Deployed Fully
Predict Risk of Patient No Shows	☐	☐	☐	☐	☐
Automate Patient Visit Scheduling	☐	☐	☐	☐	☐
AI-Enabled Triage	☐	☐	☐	☐	☐

List Other AI Tools Related to Access and Marketing and indicate Developing, Piloting, or Deploying:

11. For each of the following AI tools related to **Revenue Cycle,** please indicate the extent to which they are being developed, piloted or deployed in your health system.

	Not at All	Developing (including research)	Piloting	Deploying in Limited Area(s)	Deployed Fully
Medical Coding	☐	☐	☐	☐	☐
Automate Utilization Review	☐	☐	☐	☐	☐
Streamline Prior Authorization	☐	☐	☐	☐	☐

List Other AI Tools Related to Revenue Cycle and indicate Developing, Piloting, or Deploying:

12. For each of the following AI tools related to **Business Functions Besides Revenue Cycle**, please indicate the extent to which they are being developed, piloted or deployed in your health system.

	Not at All	Developing (including research)	Piloting	Deploying in Limited Area(s)	Deployed Fully
Forecast Census and Staffing	☐	☐	☐	☐	☐
Optimize Patient Room Utilization	☐	☐	☐	☐	☐
Optimize Operating Room Utilization	☐	☐	☐	☐	☐
Supply Chain Tool(s)	☐	☐	☐	☐	☐
Recruiting and Human Resource Tool(s)	☐	☐	☐	☐	☐
Cash Forecasting (Treasury)	☐	☐	☐	☐	☐

List Other AI Tools Related to Business Functions (Besides Revenue Cycle) and indicate Developing, Piloting, or Deploying:

13. For each of the following AI tools related to **Automating Analytics**, please indicate the extent to which they are being developed, piloted or deployed in your health system.

	Not at All	Developing (including research)	Piloting	Deploying in Limited Area(s)	Deployed Fully
Data Analysis	☐	☐	☐	☐	☐
AI-enabled computer coding	☐	☐	☐	☐	☐

List Other AI Tools Related to Automating Analytics and indicate Developing, Piloting, or Deploying:

14. For each of the following AI tools related to **Supporting Research**, please indicate the extent to which they are being developed, piloted or deployed in your health system.

	Not at All	Developing (including research)	Piloting	Deploying in Limited Area(s)	Deployed Fully
Clinical Trials Automation	☐	☐	☐	☐	☐
Drug Discovery	☐	☐	☐	☐	☐
Genetics, Genomics Tools	☐	☐	☐	☐	☐

List other AI Tools in Supporting Research and indicate developing, piloting or deploying.

15. What **other AI tools** are being developed, piloted or deployed in your health system?

Please list here and indicate whether they are in development, piloting, or deployed.

5. Success/Failure of AI Tools

16. For each type of AI use case you are piloting or deploying, please indicate **your perception of overall success to date.**

	NA (Not Piloting or Deploying)	Highly Successful	Mixed Performance	Not Working Well	Abandoned	Too Soon to Tell
Clinical Documentation	◯	◯	◯	◯	◯	◯
Clinical Chart Review	◯	◯	◯	◯	◯	◯
Clinical Risk Stratification	◯	◯	◯	◯	◯	◯
Diagnosis	◯	◯	◯	◯	◯	◯
Engage Patients in Care & Communication	◯	◯	◯	◯	◯	◯
Facilitate Patient Access & Marketing	◯	◯	◯	◯	◯	◯
Revenue Cycle	◯	◯	◯	◯	◯	◯
Business Functions outside Revenue Cycle	◯	◯	◯	◯	◯	◯
Automate Analytics	◯	◯	◯	◯	◯	◯
Support Clinical Research	◯	◯	◯	◯	◯	◯
Other AI Tools Not in the Above	◯	◯	◯	◯	◯	◯

Comments

17. For the AI tools you are developing, piloting or deploying, please indicate the extent to which you **use any of the following types of metrics** to assess AI tool performance.

	Always Measure	Sometimes Measure	Rarely Measure	Do Not Measure
AI Tool/Model Performance	◯	◯	◯	◯
Use of the AI Tool (Uptake)	◯	◯	◯	◯
Appropriate Use of the AI Tool	◯	◯	◯	◯
Patient Quality/Safety/Outcomes	◯	◯	◯	◯
Financial(including ROI)	◯	◯	◯	◯
Workflow Efficiency	◯	◯	◯	◯
Patient Health Literacy	◯	◯	◯	◯
Health Equity/Disparities	◯	◯	◯	◯
Clinician Burden/Time	◯	◯	◯	◯

What other types of measures are you using to assess AI Tools?

18. What are the **biggest barriers or obstacles** your health system has experienced in developing, piloting and deploying AI tools?

Please select all that apply and slide the answers to rank where 1=biggest obstacle.

- Financial concerns ☐ N/A
- Regulatory or compliance uncertainty ☐ N/A
- Lack of leadership support ☐ N/A
- Lack of clinician use/adoption ☐ N/A
- Insufficient expertise or technology in-house ☐ N/A
- Lack of AI tool maturity ☐ N/A

6. We Need Your Help

19. We are interested in developing a series of **case studies** of AI-deployment successes and failures. The purpose of the case studies is to help other SI members and the field learn from practical development and real-world implementation of AI tools.

Would you be willing to contribute to a case study?

- ◯ Yes!
- ◯ Not at this time
- ◯ I'd like to learn more

20. What ***topics*** do you think the Scottsdale Institute should focus on regarding using AI tools in practice?

Thank You for Participating in this Survey!
We look forward to sharing the results with you.

Appendix 3 – Metrics Used to Assess AI Performance

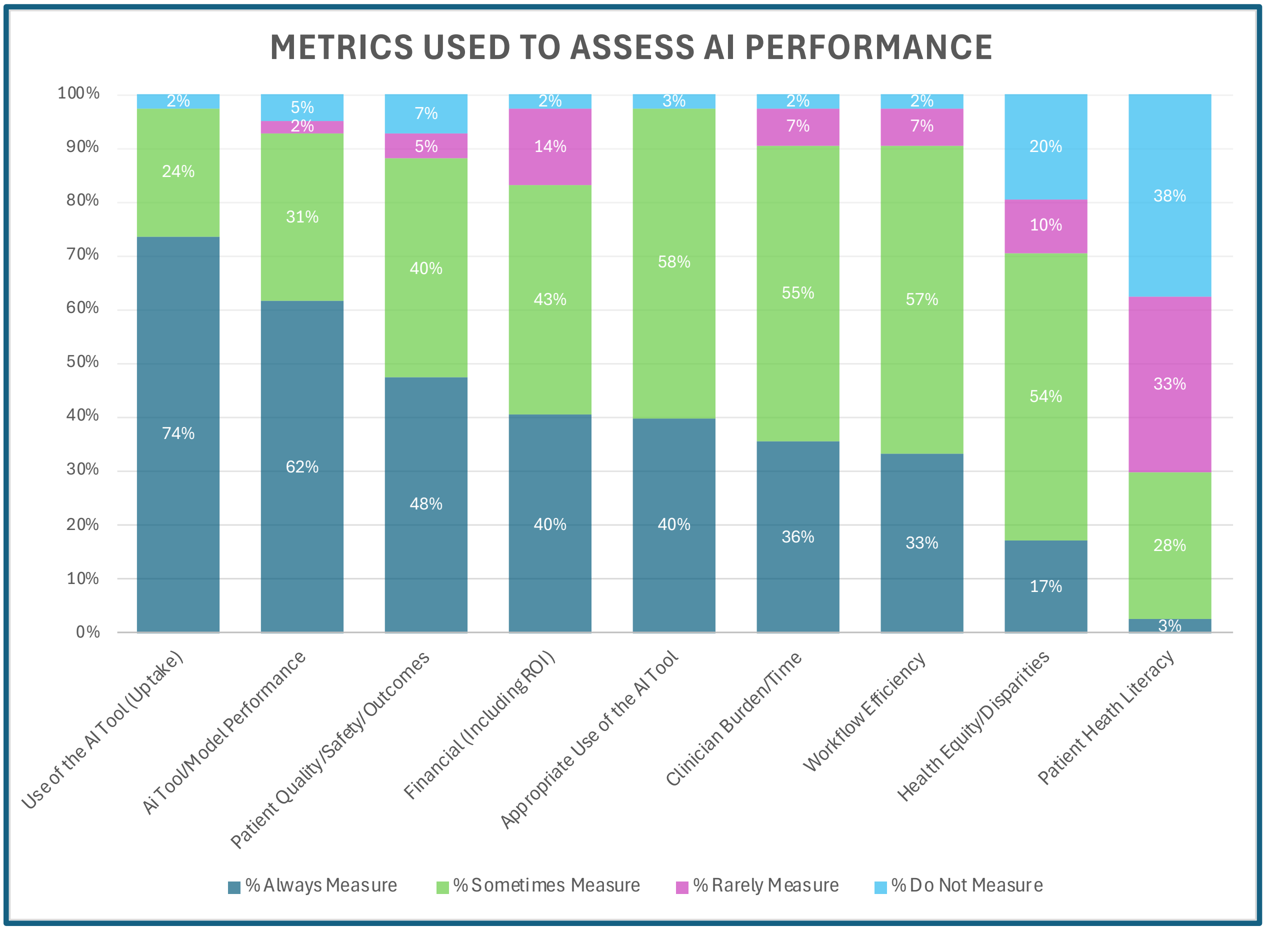

Supplement: ocaf065_Supplementary_Data [file ocaf065_supplementary_data.pdf]
